# Supplementary material for: HIV-1 Tat favors the multiplication of Mycobacterium tuberculosis and Toxoplasma by inhibiting clathrin-mediated endocytosis and autophagy
Source: PLoS Pathog. 2025 Sep 11;21(9):e1013183. doi: 10.1371/journal.ppat.1013183 (PMC12445553; doi:10.1371/journal.ppat.1013183)
Supplement: S1 Fig — hMDMs were treated with 15 nM Tat (or mutants) for 5 h before harvesting supernatant for cytokine assays. Data are means ± SEM (n = 3) of cells from three different donors. One-way ANOVA compared to Tat WT (*, p < 0.05; **, p < 0.01). (PDF) [file ppat.1013183.s001.pdf]

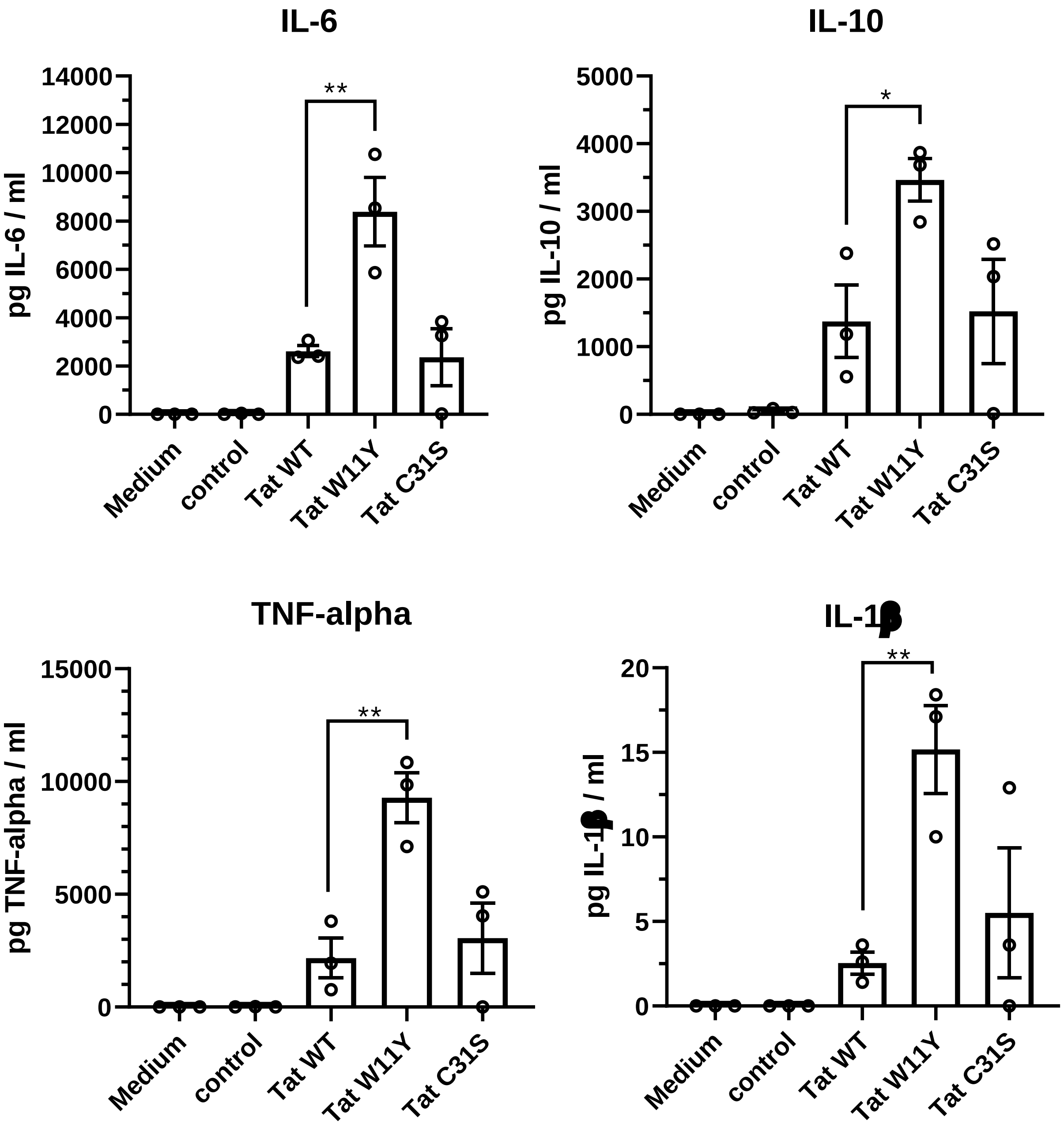

S1 Fig. Tat WT, Tat-W11Y and Tat-C31S induce cytokine secretion. hMDMs were treated with 15 nM Tat (or mutants) for 5 h before harvesting supernatant for cytokine assays. Data are means ± SEM (n=3) of cells from three different donors. One-way ANOVA compared to Tat WT (*, $p<0.05$; **, $p<0.01$).
