# Supplementary material for: HIV-1 Tat favors the multiplication of Mycobacterium tuberculosis and Toxoplasma by inhibiting clathrin-mediated endocytosis and autophagy
Source: PLoS Pathog. 2025 Sep 11;21(9):e1013183. doi: 10.1371/journal.ppat.1013183 (PMC12445553; doi:10.1371/journal.ppat.1013183)
Supplement: S2 Fig — RAW 264.7 cells were transfected with a vector containing the Firefly gene behind 3 NF-KB sites, and another expressing the Renilla luciferase. Cells were then treated for 5 h with 15 nM Tat (WT, W11Y or C31S as indicated) or 0.5 µg/ ml of E coli LPS as positive control, before harvesting cells for luciferase assays. Results (means ± SEM, n = 3 independent experiments) are expressed as Firefly/ Renilla activity ratio. One-way ANOVA compared to control (*, p < 0.05). (PDF) [file ppat.1013183.s002.pdf]

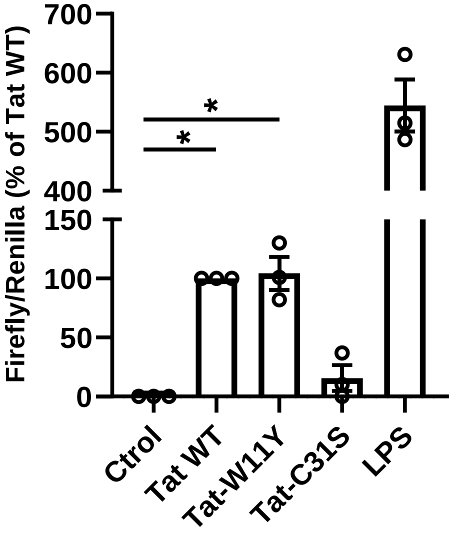

S2 Fig. Tat WT and Tat-W11Y but not Tat-C31S activate NF-KB. RAW 264.7 cells were transfected with a vector containing the Firefly gene behind 3 NF-KB sites, and another expressing the Renilla luciferase. Cells were then treated for 5 h with 15 nM Tat (WT, W11Y or C31S as indicated) or 0.5 µg/ ml of E coli LPS as positive control, before harvesting cells for luciferase assays. Results (means ± SEM, n=3 independent experiments) are expressed as Firefly / Renilla activity ratio. One-way ANOVA compared to control (*, $p<0.05$).
