# Supplementary material for: HIV-1 Tat favors the multiplication of Mycobacterium tuberculosis and Toxoplasma by inhibiting clathrin-mediated endocytosis and autophagy
Source: PLoS Pathog. 2025 Sep 11;21(9):e1013183. doi: 10.1371/journal.ppat.1013183 (PMC12445553; doi:10.1371/journal.ppat.1013183)
Supplement: S3 Fig — Zebrafish embryos (n = 20–25 for each group) at 24 hpf were injected with Tat (~100 nM final concentration), WT or C31S as indicated. Injections were repeated at day 2,3,4 and 5. (PDF) [file ppat.1013183.s003.pdf]

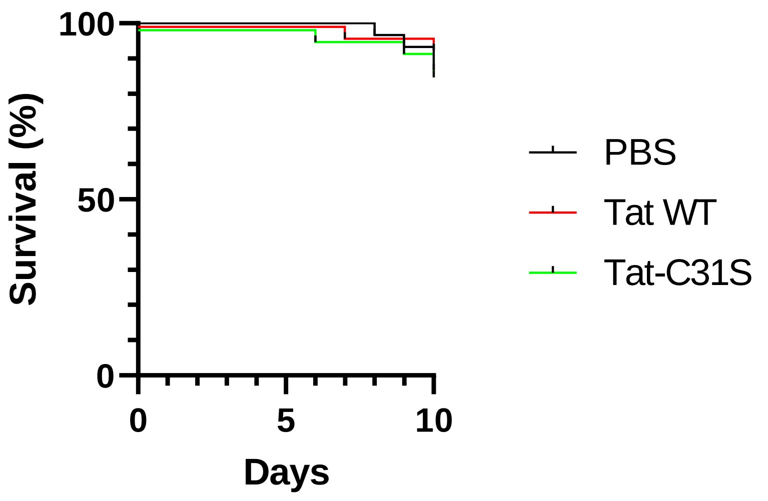

S3 Fig. Tat injections do not affect the survival of zebrafish embryos. Zebrafish embryos (n=20-25 for each group) at 24 hpf were injected with Tat (~100 nM final concentration), WT or C31S as indicated. Injections were repeated at day 2,3,4 and 5.
