# Supplementary material for: HIV-1 Tat favors the multiplication of Mycobacterium tuberculosis and Toxoplasma by inhibiting clathrin-mediated endocytosis and autophagy
Source: PLoS Pathog. 2025 Sep 11;21(9):e1013183. doi: 10.1371/journal.ppat.1013183 (PMC12445553; doi:10.1371/journal.ppat.1013183)
Supplement: S4 Fig — A, Zebrafish embryos (n = 20–25 for each group) at 24 hpf were injected with Tat (~100 nM final concentration) then infected with Mycobacterium marinum. Tat injection (WT or W11Y) was repeated at dpi 2,3,4 and 5. Survival curves of the Tat groups (WT/ W11Y) were compared using Log-rank Test (**, p < 0.01). B, zebrafishes were infected with M. marinum expressing tdTomato, and granulomas were counted after the indicated number of dpi. Two ways ANOVA (**, p < 0.01). (PDF) [file ppat.1013183.s004.pdf]

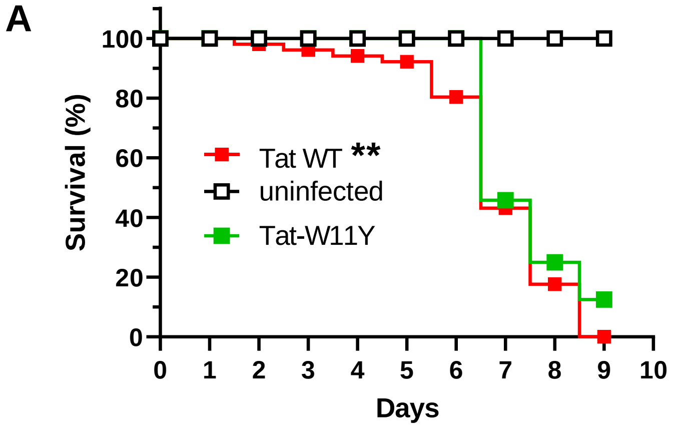

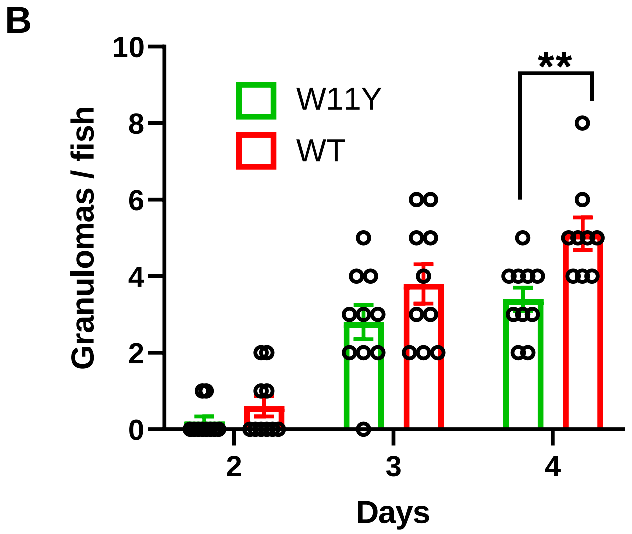

S4 Fig. Effect of Tat W11Y on the multiplication of *Mycobacterium marinum* in zebrafishes. A, Zebrafish embryos (n=20-25 for each group) at 24 hpf were injected with Tat (~100 nM final concentration) then infected with *Mycobacterium marinum.* Tat injection (WT or W11Y) was repeated at dpi 2,3,4 and 5. Survival curves of the Tat groups (WT/ W11Y) were compared using Log-rank Test (**, p<0.01). B, zebrafishes were infected with *M. marinum* expressing tdTomato, and granulomas were counted after the indicated number of dpi. Two ways ANOVA (**, p<0.01).
