# Supplementary material for: HIV-1 Tat favors the multiplication of Mycobacterium tuberculosis and Toxoplasma by inhibiting clathrin-mediated endocytosis and autophagy
Source: PLoS Pathog. 2025 Sep 11;21(9):e1013183. doi: 10.1371/journal.ppat.1013183 (PMC12445553; doi:10.1371/journal.ppat.1013183)
Supplement: S5 Fig — Zebrafish embryos at 24 hpf were infected with Mycobacterium marinum expressing tdTomato, and imaged at 3 dpi. Arrows point to granulomas. Bar, 200 µm. (PDF) [file ppat.1013183.s005.pdf]

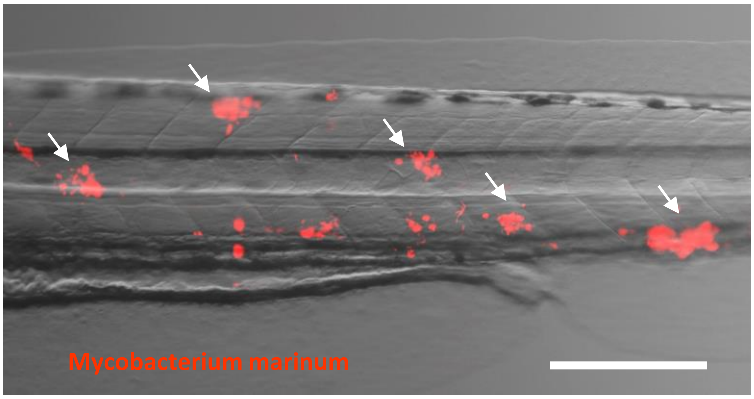

S5 Fig. Visualisation of granulomas in Zebrafish embryos. Zebrafish embryos at 24 hpf were infected with *Mycobacterium marinum* expressing tdTomato, and imaged at 3 dpi. Arrows point to granulomas. Bar, 200 µm.
