# Supplementary material for: HIV-1 Tat favors the multiplication of Mycobacterium tuberculosis and Toxoplasma by inhibiting clathrin-mediated endocytosis and autophagy
Source: PLoS Pathog. 2025 Sep 11;21(9):e1013183. doi: 10.1371/journal.ppat.1013183 (PMC12445553; doi:10.1371/journal.ppat.1013183)
Supplement: S6 Fig — hMDMs were pretreated or not with 15 nM Tat for 5 h, then infected with opsonized T. gondii (MOI = 10) for 30 min before staining for opsonizing antibody and GRA3, then DAPI staining. Representative confocal sections are shown. Bar, 10 µm. Three different staining patterns were observed: parasite surface labelled by opsonizing Ab only (I), parasitophorous vacuole membrane staining by GRA3 only (arrow, II) or labeling with the opsonizing Ab and some dotty GRA3 staining not delineating the whole parasitophorous membrane periphery (III). The graph shows the quantification (mean ± SEM) for 100–150 parasites counted on two coverslips for each condition. (PDF) [file ppat.1013183.s006.pdf]

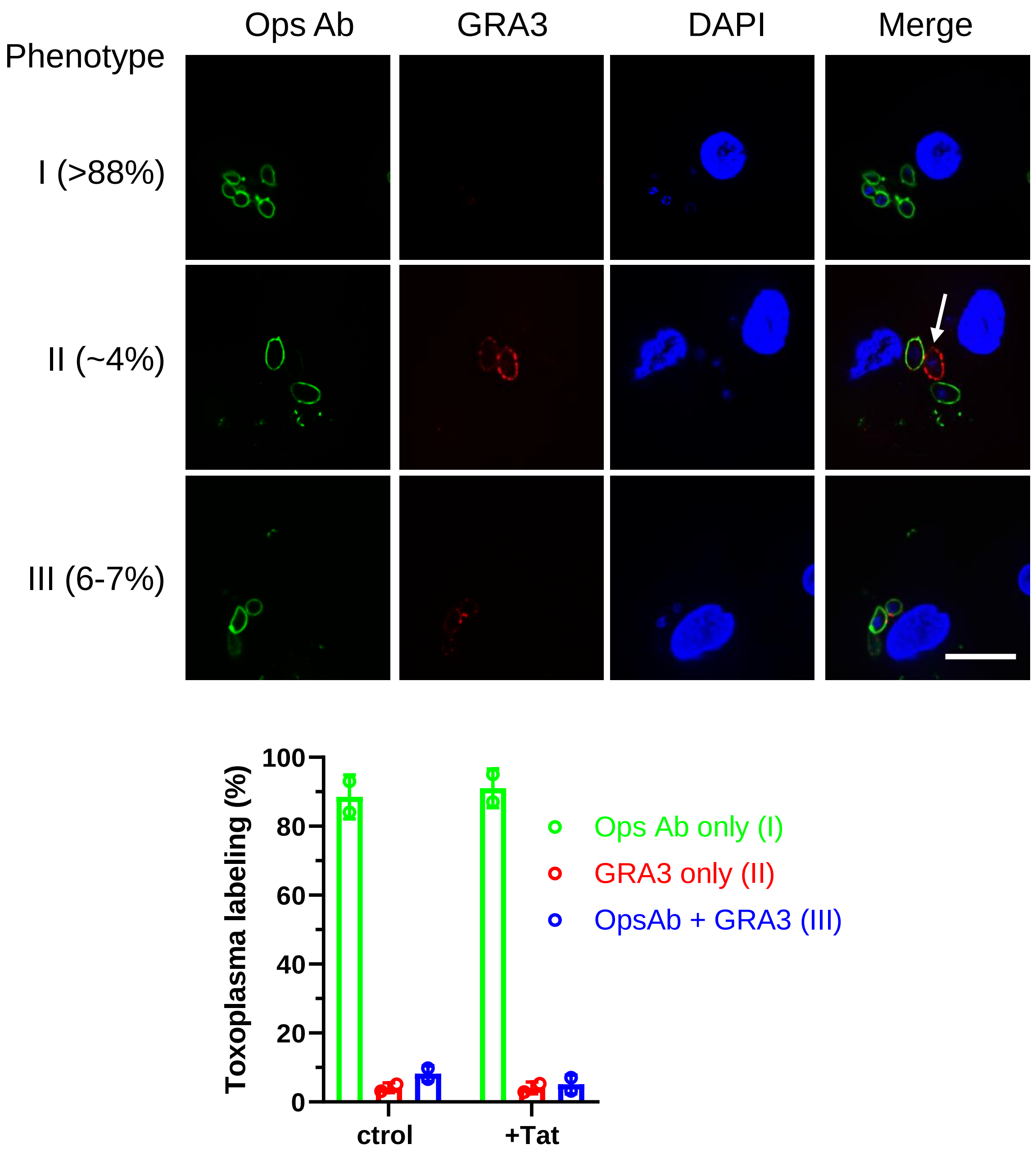

S6 Fig. Opsonization of *T. gondii* induces entry of the parasite by phagocytosis. hMDMs were pretreated or not with 15 nM Tat for 5 h, then infected with opsonized T. gondii (MOI=10) for 30 min before staining for opsonizing antibody and GRA3, then DAPI staining. Representative confocal sections are shown. Bar, 10 µm. Three different staining patterns were observed : parasite surface labelled by opsonizing Ab only (I), parasitophorous vacuole membrane staining by GRA3 only (arrow, II) or labeling with the opsonizing Ab and some dotty GRA3 staining not delineating the whole parasitophorous membrane periphery (III). The graph shows the quantification (mean ± SEM) for 100-150 parasites counted on two coverslips for each condition.
