# Supplementary material for: HIV-1 Tat favors the multiplication of Mycobacterium tuberculosis and Toxoplasma by inhibiting clathrin-mediated endocytosis and autophagy
Source: PLoS Pathog. 2025 Sep 11;21(9):e1013183. doi: 10.1371/journal.ppat.1013183 (PMC12445553; doi:10.1371/journal.ppat.1013183)
Supplement: S7 Fig — hMDMs were pretreated with 15 nM Tat (WT or mutant) for 5 h, then infected with opsonized T. gondii (MOI = 10) for 30 min before staining for p62 and opsonizing antibody, and DAPI staining. Representative confocal sections are shown. Bar, 10 µm. The graph shows the quantification of the fraction of p62+ Toxoplasma on 250–500 parasites. One Way ANOVA, ****, p < 0.0001. (PDF) [file ppat.1013183.s007.pdf]

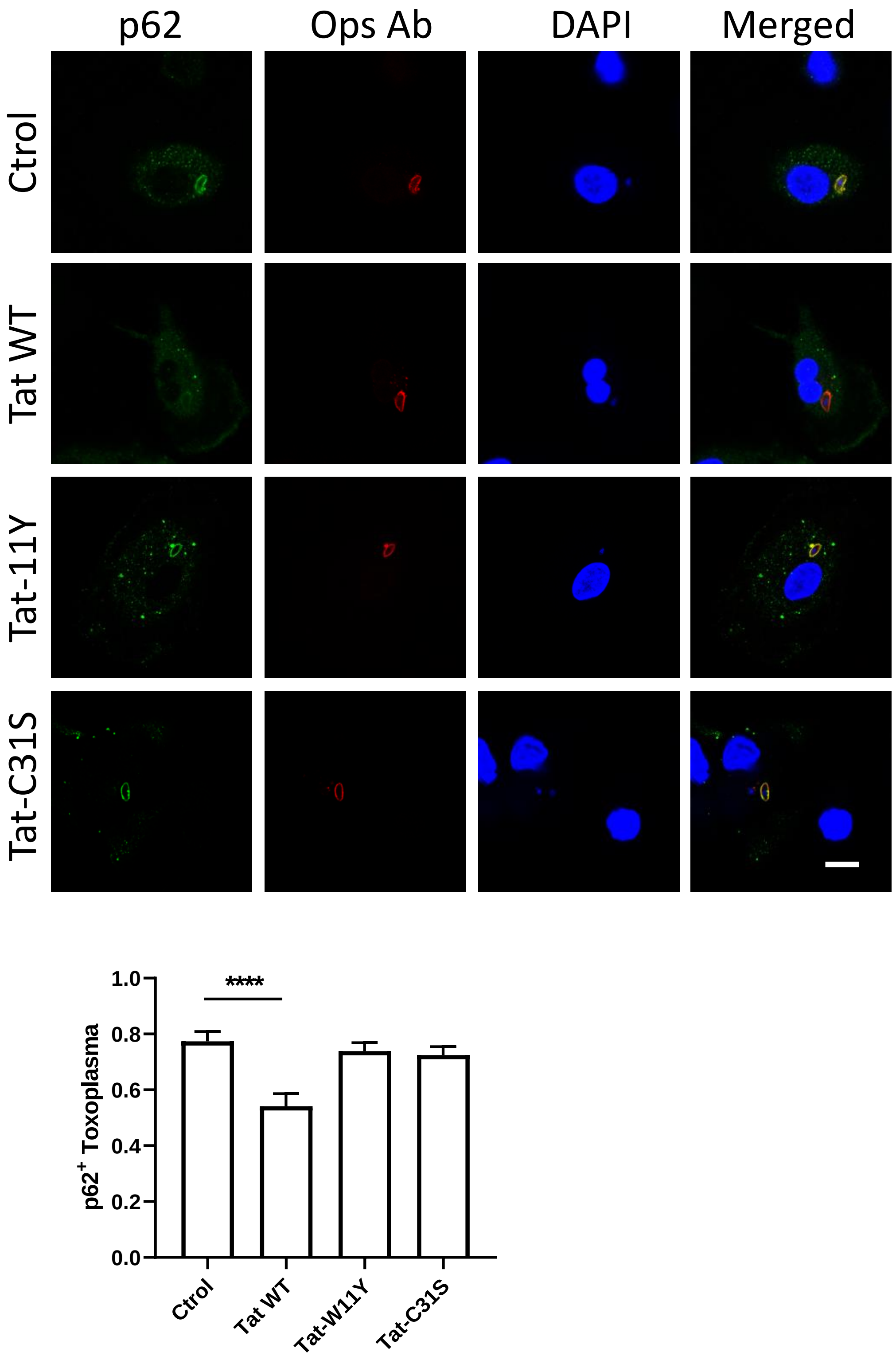

S7 Fig. Tat inhibits the recruitment of p62 on vacuoles containing *T. gondii*. hMDMs were pretreated with 15 nM Tat (WT or mutant) for 5 h, then infected with opsonized T. gondii (MOI=10) for 30 min before staining for p62 and opsonizing antibody, and DAPI staining. Representative confocal sections are shown. Bar, 10 µm. The graph shows the quantification of the fraction of $p62^+$ toxoplasma on 250-500 parasites. One Way ANOVA, ****, p<0.00001.
