# Supplementary material for: HIV-1 Tat favors the multiplication of Mycobacterium tuberculosis and Toxoplasma by inhibiting clathrin-mediated endocytosis and autophagy
Source: PLoS Pathog. 2025 Sep 11;21(9):e1013183. doi: 10.1371/journal.ppat.1013183 (PMC12445553; doi:10.1371/journal.ppat.1013183)
Supplement: S8 Fig — RAW macrophages were transfected with mCherry-LC3 and the indicated version of Tat. The Tat expression vector is bicistronic and also expresses EGFP. Cells were fixed after 18h and imaged using a spinning disk confocal microscope and a x100 NA 1.45 objective. Bar, 10 µm. Less autophagosomes were present in cells transfected with Tat WT and Tat(55–57)A. (PDF) [file ppat.1013183.s008.pdf]

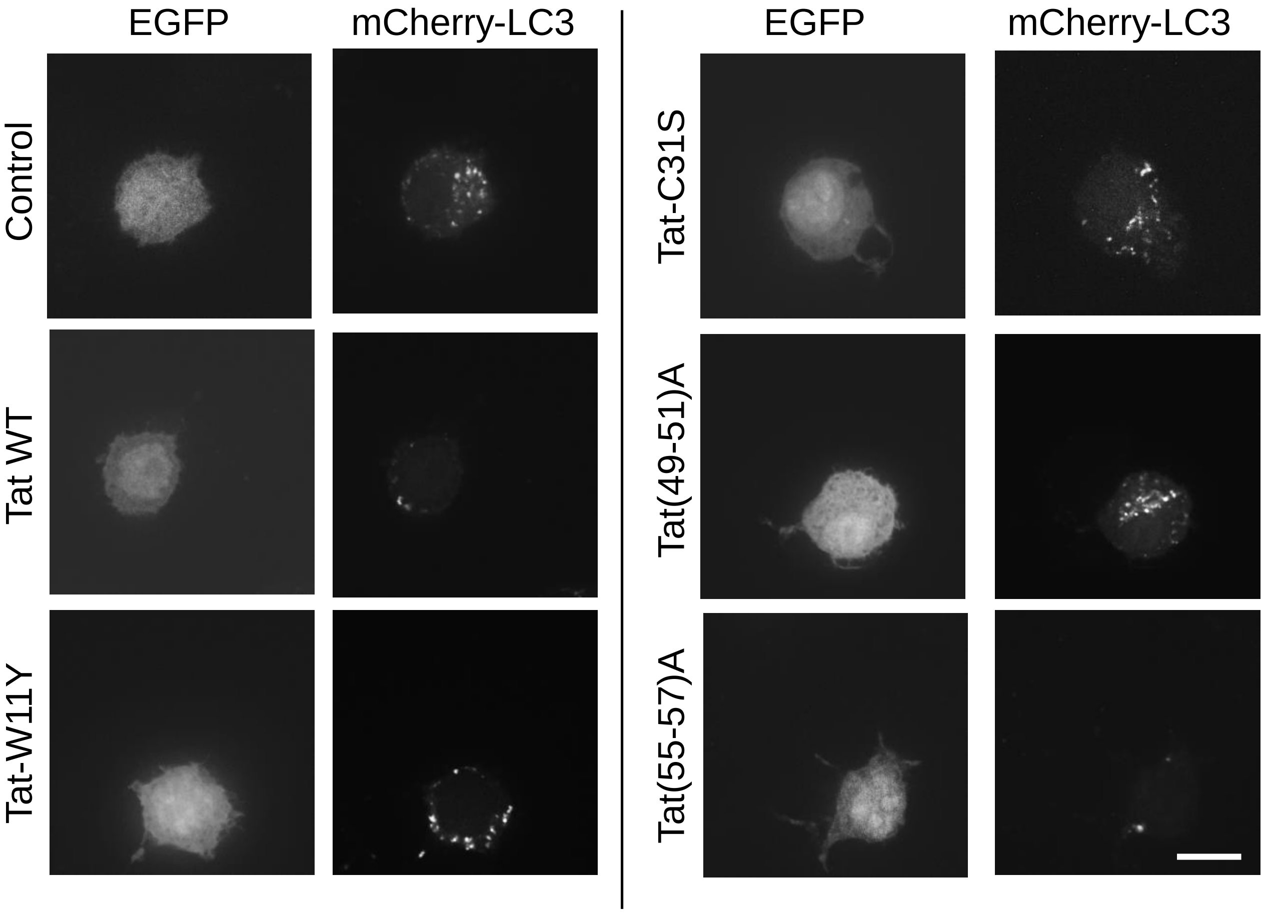

S8 Fig. Effect of Tat mutants on autophagosome numbers. RAW macrophages were transfected with mCherry-LC3 and the indicated version of Tat. The Tat expression vector is bicistronic and also expresses EGFP. Cells were fixed after 18h and imaged using a spinning disk confocal microscope and a x100 NA 1.45 objective. Bar, 10 µm. Less autophagosomes were present in cells transfected with Tat WT and Tat(55-57)A.
