# Supplementary material for: HIV-1 Tat favors the multiplication of Mycobacterium tuberculosis and Toxoplasma by inhibiting clathrin-mediated endocytosis and autophagy
Source: PLoS Pathog. 2025 Sep 11;21(9):e1013183. doi: 10.1371/journal.ppat.1013183 (PMC12445553; doi:10.1371/journal.ppat.1013183)
Supplement: S9 Fig — RAW macrophages were transfected with mRFP-EGFP-LC3 then treated with 15 nM Tat for 4 h. When indicated, 20 nM bafilomycin A1 was added for 1 h and cells were imaged at 37°C using a spinning-disk confocal microscope. Bar, 10 µm. Green structures accumulated when bafilomycin was present. (PDF) [file ppat.1013183.s009.pdf]

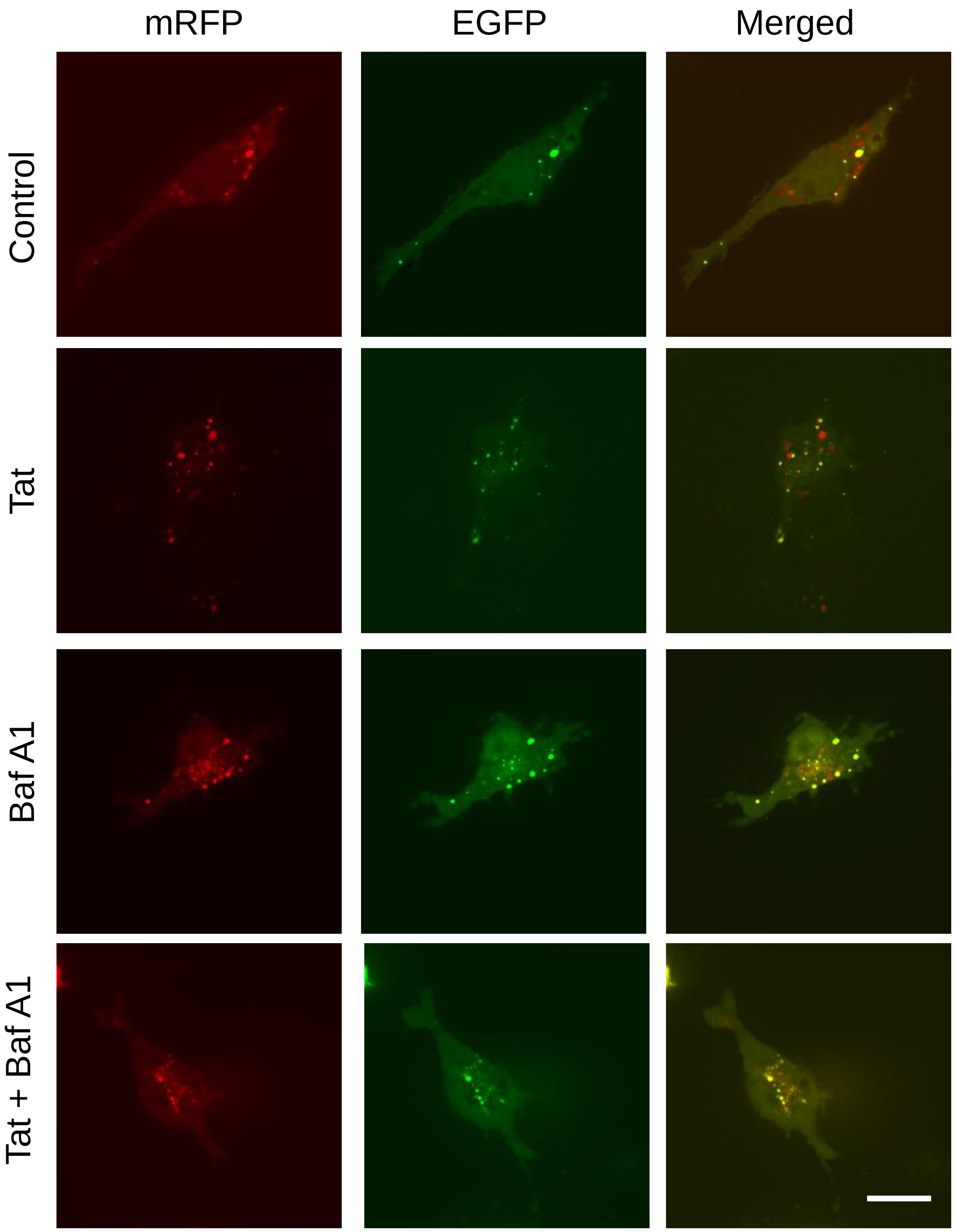


**S9 Fig. Tat does not affect the autophagic flux, as monitored using mRFP-EGFP-LC3.** RAW macrophages were transfected with mRFP-EGFP-LC3B then treated with 15 nM Tat for 4 h. When indicated, 20 nM Bafilomycin A1 was added for 1 h and cells were imaged at 37°C using a spinning-disk confocal microscope. Bar, 10 µm. Green structures accumulated when Baf was present.
