# Supplementary material for: HIV-1 Tat favors the multiplication of Mycobacterium tuberculosis and Toxoplasma by inhibiting clathrin-mediated endocytosis and autophagy
Source: PLoS Pathog. 2025 Sep 11;21(9):e1013183. doi: 10.1371/journal.ppat.1013183 (PMC12445553; doi:10.1371/journal.ppat.1013183)
Supplement: S10 Fig — RAW cells were cotransfected with AP-2σ2-EGFP and the indicated siRNA before fixation and imaging by TIRF microscopy using a x100 NA and a 488 nm laser for EGFP fluorescence, or a LED for transmitted light (TRANS). Bar, 10 µm. (PDF) [file ppat.1013183.s010.pdf]

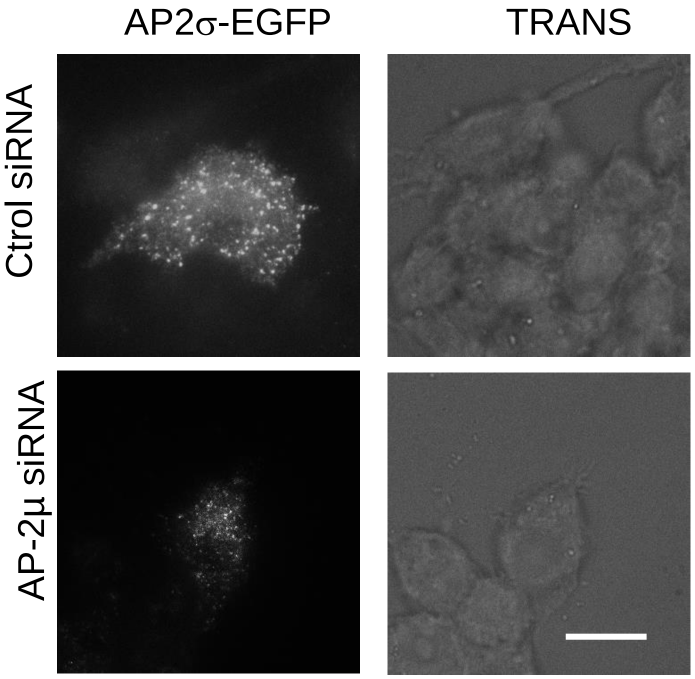

S10 Fig. AP-2µ siRNA inhibits AP-2σ2-EGFP recruitment at the plasma membrane. RAW cells were cotransfected with AP-2σ2-EGFP and the indicated siRNA before fixation and imaging by TIRF microscopy using a x100 NA and a 488 nm laser for EGFP fluorescence, or a LED for transmitted light (TRANS). Bar, 10 µm.
