# Supplementary material for: HIV-1 Tat favors the multiplication of Mycobacterium tuberculosis and Toxoplasma by inhibiting clathrin-mediated endocytosis and autophagy
Source: PLoS Pathog. 2025 Sep 11;21(9):e1013183. doi: 10.1371/journal.ppat.1013183 (PMC12445553; doi:10.1371/journal.ppat.1013183)
Supplement: S11 Fig — RAW macrophages were transfected with AP-2σ2-EGFP, treated with 15 nM of the indicated Tat mutant for 5h before fixation and imaging by TIRF microscopy using a 488 nm laser for EGFP fluorescence or a LED for transmitted light (TRANS). Bar, 10 µm. (PDF) [file ppat.1013183.s011.pdf]

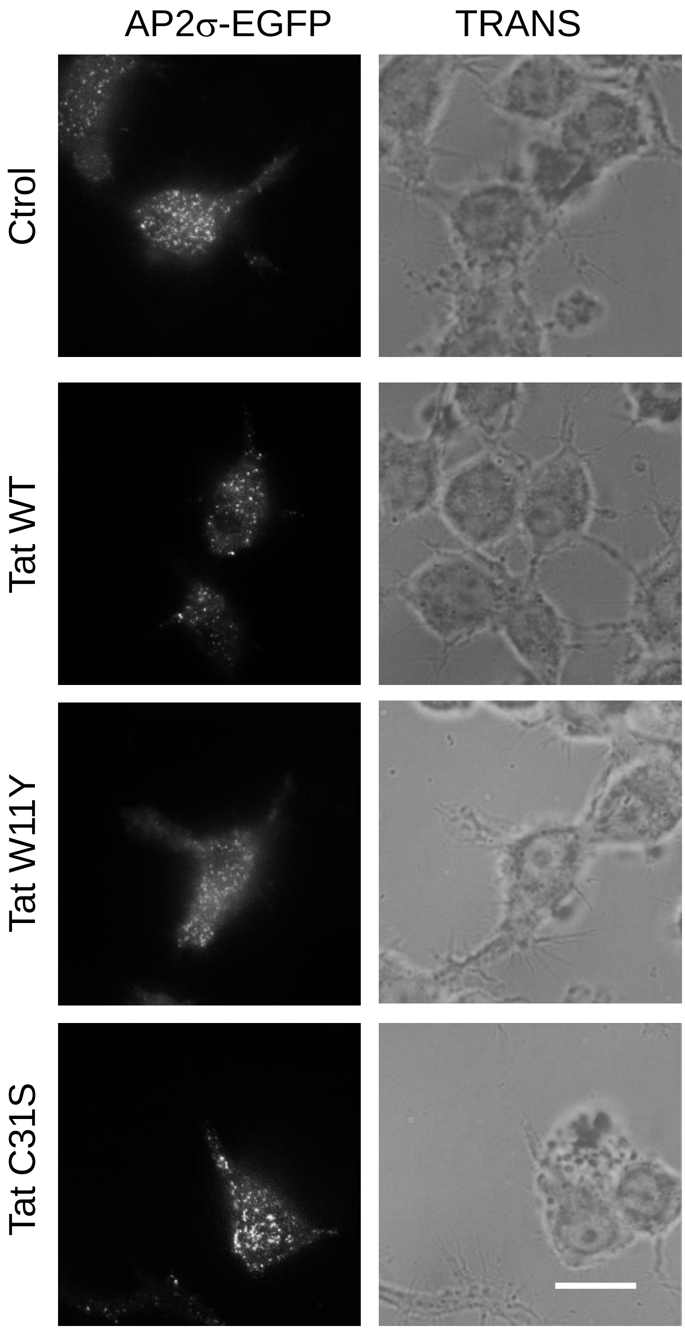

S11 Fig. Tat palmitoylation is required for Tat to inhibit AP-2 recruitment. RAW macrophages were transfected with AP-2σ2-EGFP, treated with 15 nM of the indicated Tat mutant for 5h before fixation and imaging by TIRF microscopy using a 488 nm laser for EGFP fluorescence or a LED for transmitted light (TRANS). Bar, 10 µm.
