# Supplementary material for: HIV-1 Tat favors the multiplication of Mycobacterium tuberculosis and Toxoplasma by inhibiting clathrin-mediated endocytosis and autophagy
Source: PLoS Pathog. 2025 Sep 11;21(9):e1013183. doi: 10.1371/journal.ppat.1013183 (PMC12445553; doi:10.1371/journal.ppat.1013183)
Supplement: S12 Fig — RAW 264.7 macrophages were cotransfected with AP-2σ2-EGFP and the indicated Tat mutant before fixation and imaging by TIRF microscopy. Bar, 5 µm. (PDF) [file ppat.1013183.s012.pdf]

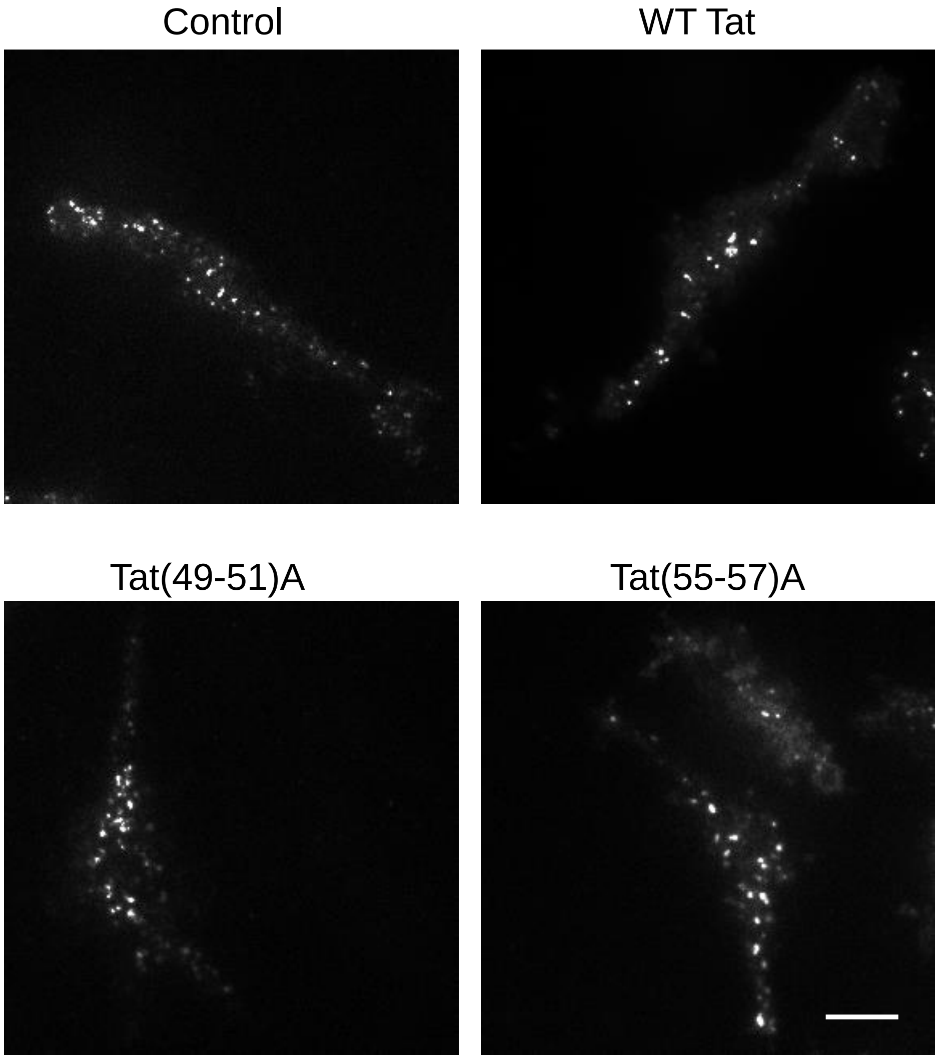

S12 Fig. Tat Binding to PI(4,5)P2 is required for Tat to inhibit AP-2 recruitment. RAW 264.7 macrophages were cotransfected with AP-2σ2-EGFP and the indicated Tat mutant before fixation and imaging by TIRF microscopy. Bar, 5 µm.
