# Supplementary material for: HIV-1 Tat favors the multiplication of Mycobacterium tuberculosis and Toxoplasma by inhibiting clathrin-mediated endocytosis and autophagy
Source: PLoS Pathog. 2025 Sep 11;21(9):e1013183. doi: 10.1371/journal.ppat.1013183 (PMC12445553; doi:10.1371/journal.ppat.1013183)
Supplement: S13 Fig — RAW macrophages were cotransfected with AP-2σ2-EGFP, Tat and the mouse PIP-5 kinase as indicated before fixation and imaging by TIRF microscopy. Bar, 5 µm. (PDF) [file ppat.1013183.s013.pdf]

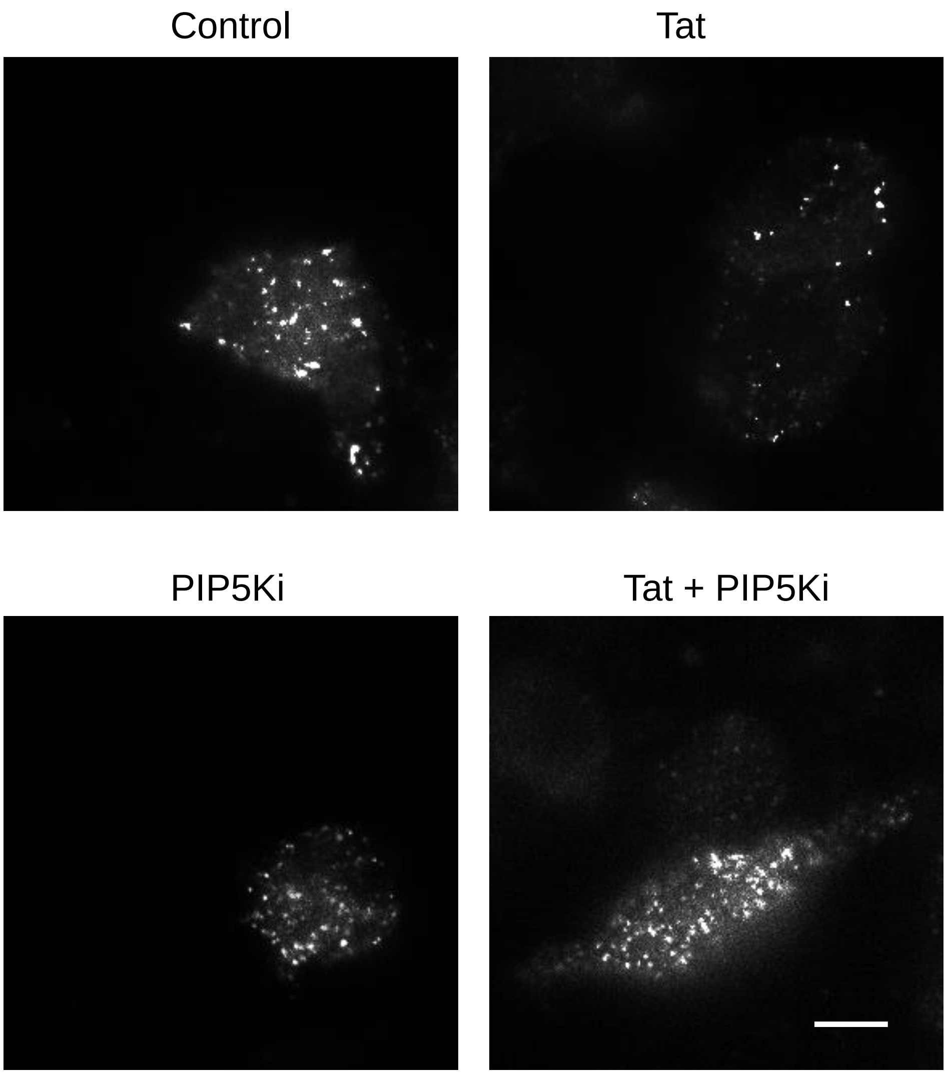

S13 Fig. Increasing PI(4,5)P2 level prevents Tat from inhibiting AP-2 recruitment. RAW macrophages were cotransfected with AP-2σ2-EGFP, Tat and the mouse PIP-5 kinase as indicated before fixation and imaging by TIRF microscopy. Bar, 5 µm.
