# Supplementary material for: HIV-1 Tat favors the multiplication of Mycobacterium tuberculosis and Toxoplasma by inhibiting clathrin-mediated endocytosis and autophagy
Source: PLoS Pathog. 2025 Sep 11;21(9):e1013183. doi: 10.1371/journal.ppat.1013183 (PMC12445553; doi:10.1371/journal.ppat.1013183)
Supplement: S14 Fig — RAW 264.7 macrophages were transfected using RNAiMAX with a control siRNA, or an siRNA against Cdc42. After 24h, cells were lysed before western blotting against Cdc42 or a-tubulin as a loading control. (PDF) [file ppat.1013183.s014.pdf]

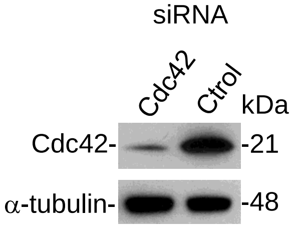

S14 Fig. An siRNA against Cdc42 inhibits its expression. RAW 264.7 macrophages were transfected using RNAiMAX with a control siRNA, or an siRNA against Cdc42. After 24h, cells were lysed before western blotting against Cdc42 or α-tubulin as a loading control.
