# Supplementary material for: HIV-1 Tat favors the multiplication of Mycobacterium tuberculosis and Toxoplasma by inhibiting clathrin-mediated endocytosis and autophagy
Source: PLoS Pathog. 2025 Sep 11;21(9):e1013183. doi: 10.1371/journal.ppat.1013183 (PMC12445553; doi:10.1371/journal.ppat.1013183)
Supplement: S15 Fig — RAW macrophages were transfected with mCherry-LC3 and Tat as indicated, then treated with 30 µM Pistop2 or Dyngo4a for 90 min before labeling cells with Cy5-transferrin for 30 min, fixation, and confocal microscopy. Bar, 10 µm. (PDF) [file ppat.1013183.s015.pdf]

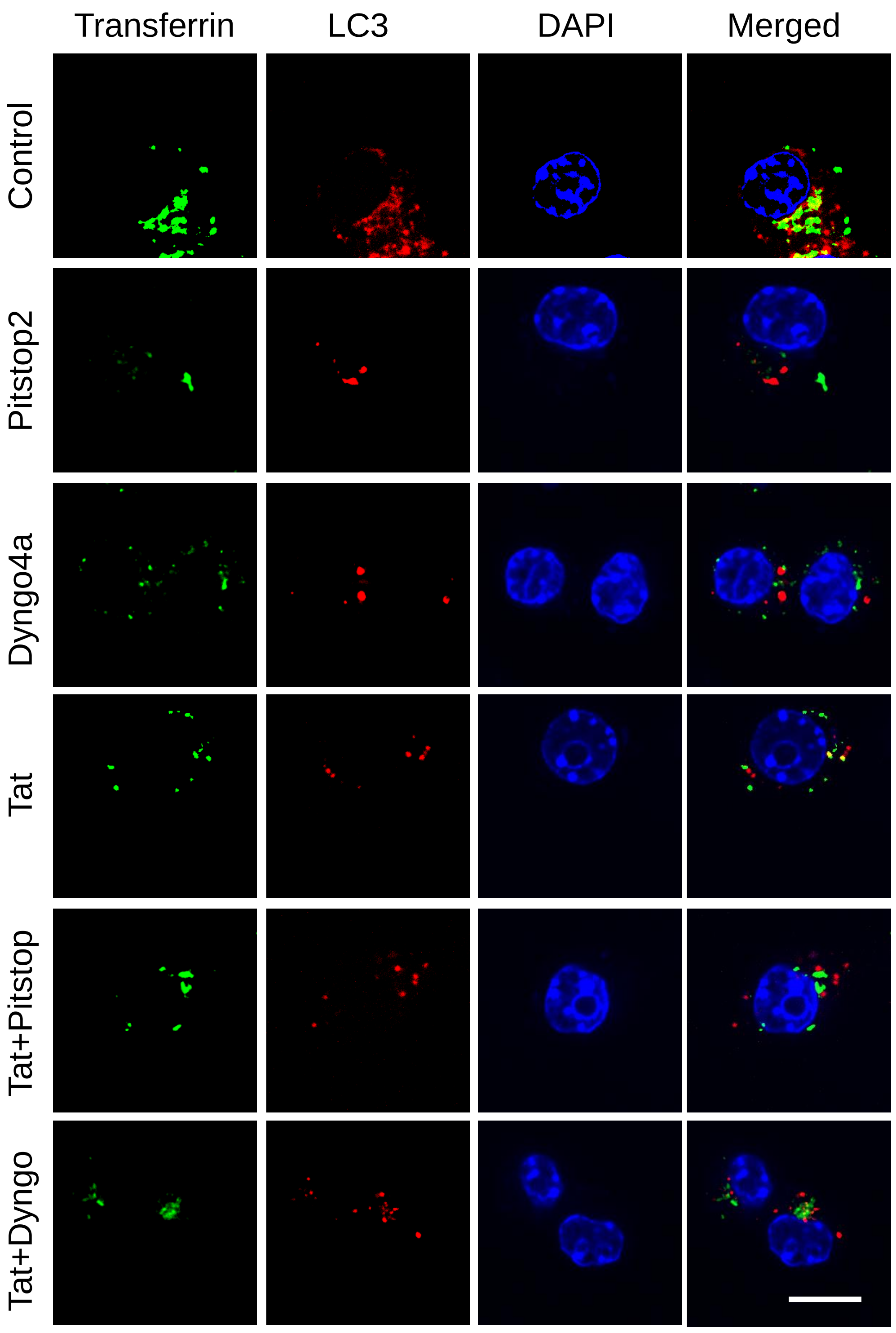

S15 Fig. CME inhibitors and Tat do not show additive inhibitory effects on CME and autophagy. RAW macrophages were transfected with mCherry-LC3 and Tat as indicated, then treated with 30 µM Pistop2 or Dyngo4a for 90 min before labeling cells with Cy5-transferrin for 30 min, fixation, and confocal microscopy. Bar, 10 µm.
