# Supplementary material for: HIV-1 Tat favors the multiplication of Mycobacterium tuberculosis and Toxoplasma by inhibiting clathrin-mediated endocytosis and autophagy
Source: PLoS Pathog. 2025 Sep 11;21(9):e1013183. doi: 10.1371/journal.ppat.1013183 (PMC12445553; doi:10.1371/journal.ppat.1013183)
Supplement: S16 Fig — RAW cells were transfected with mCherry-LC3, Dynamin2 (WT or K44A) and Tat as indicated before labeling cells with Cy5-transferrin for 30 min, fixation, and confocal microscopy. Arrow points at transfected cell when needed. Bar, 10 µm. (PDF) [file ppat.1013183.s016.pdf]

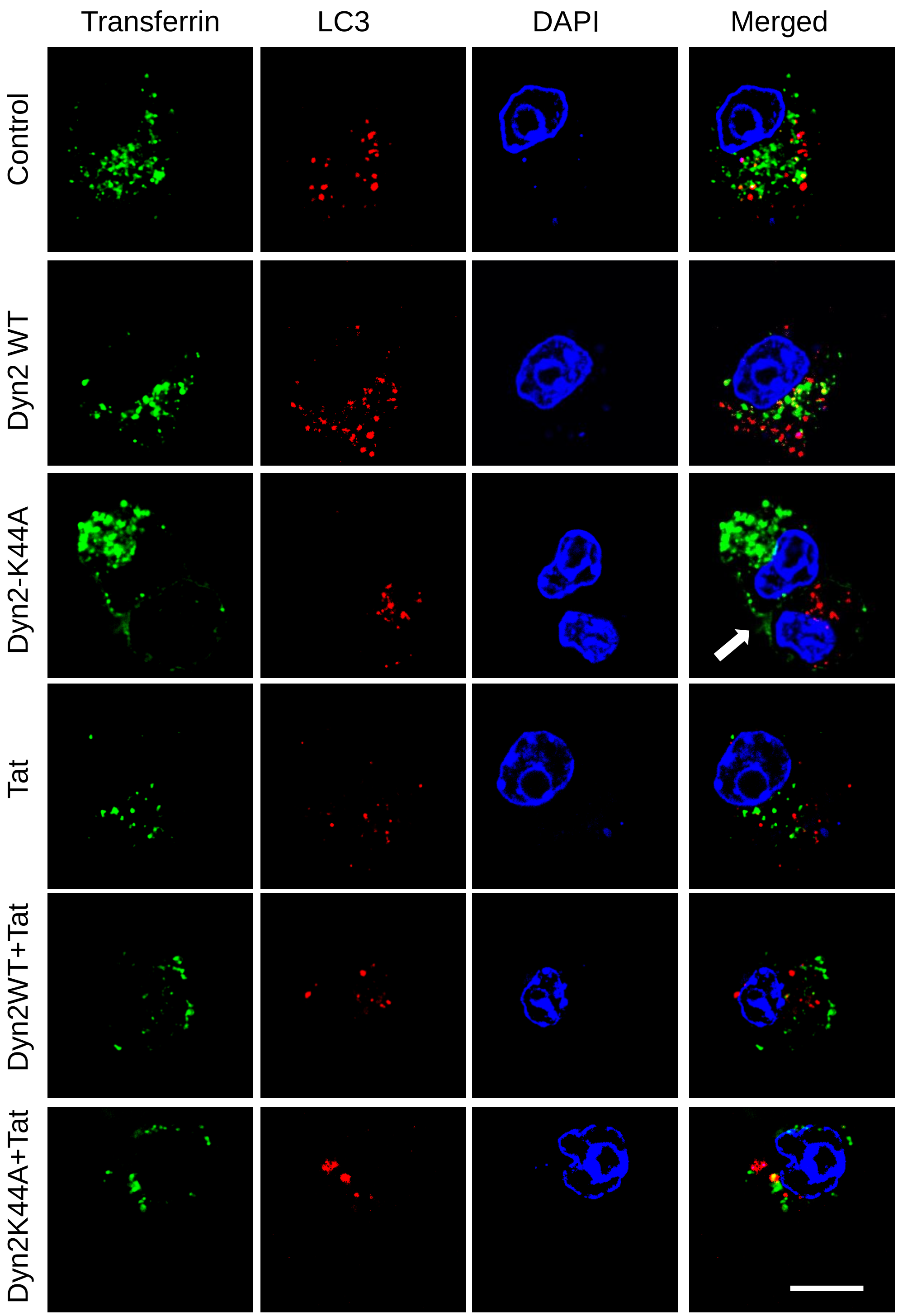

S16 Fig. Dominant-negative dynamin2 and Tat do not show additive inhibitory effects on CME and autophagy. RAW cells were transfected with mCherry-LC3, Dynamin2 (WT or K44A) and Tat as indicated before labeling cells with Cy5-transferrin for 30 min, fixation, and confocal microscopy. Arrow points at transfected cell when needed. Bar, 10 µm.
