# Supplementary material for: HIV-1 Tat favors the multiplication of Mycobacterium tuberculosis and Toxoplasma by inhibiting clathrin-mediated endocytosis and autophagy
Source: PLoS Pathog. 2025 Sep 11;21(9):e1013183. doi: 10.1371/journal.ppat.1013183 (PMC12445553; doi:10.1371/journal.ppat.1013183)
Supplement: S18 Fig — RAW 264.7 cells were transfected with mCherry-LC3, treated with 15 nM of the indicated Tat mutant for 5 h or 100 nM bafilomycin A1 for 2 h before fixation, staining with BODIPY 493/503 and DAPI, and imaging by confocal microscopy. Bar, 10 µm. (PDF) [file ppat.1013183.s018.pdf]

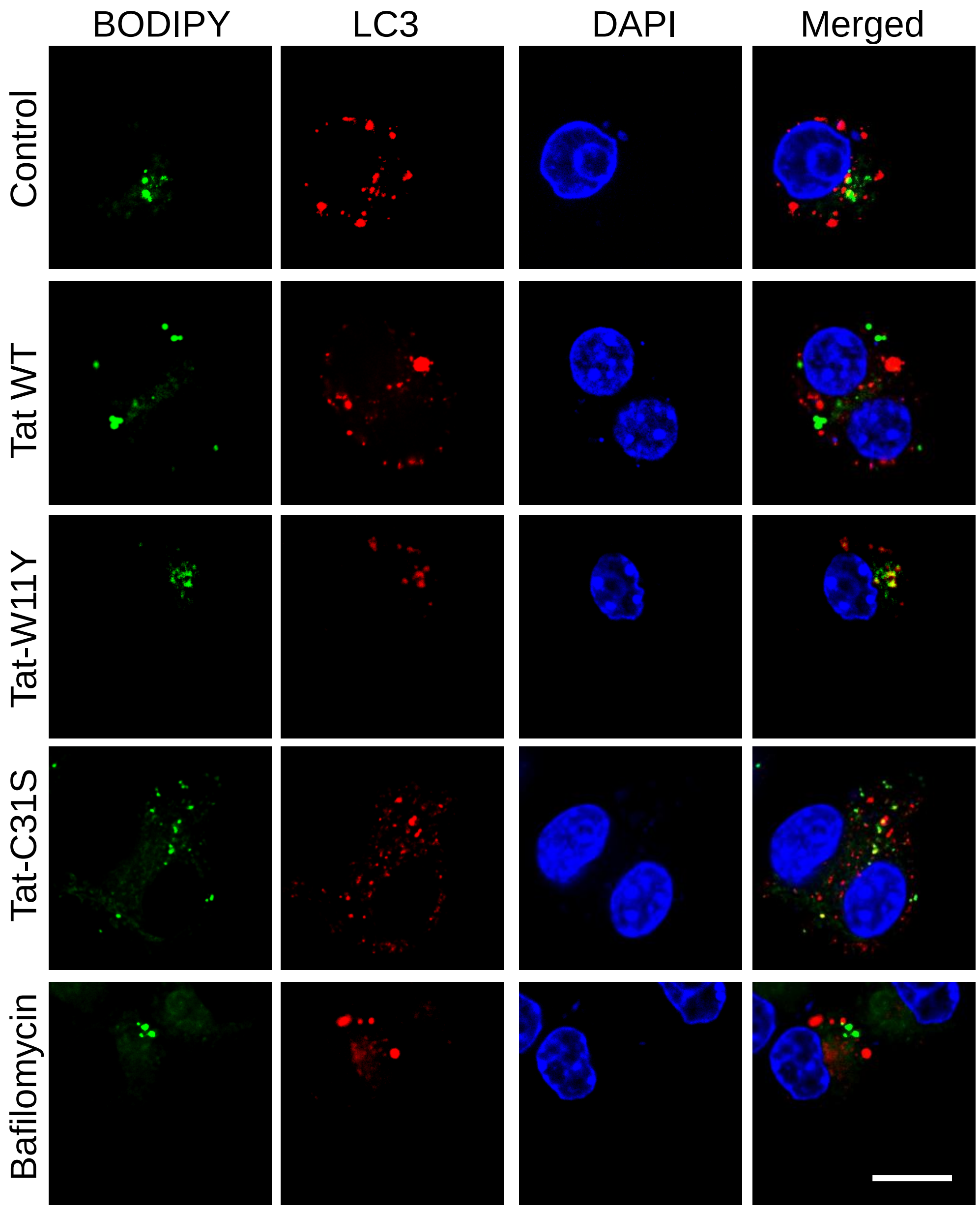

S18 Fig. Tat decreases the localization of lipid droplets in LC3-positive structures. RAW 264.7 cells were transfected with mCherry-LC3, treated with 15 nM of the indicated Tat mutant for 5 h or 100 nM bafilomycin A1 for 2 h before fixation, staining with BODIPY 493/503 and DAPI, and imaging by confocal microscopy. Bar, 10 µm.
